# Entity linking for biomedical literature

**DOI:** 10.1186/1472-6947-15-S1-S4

**Published:** 2015-05-20

**Authors:** Jin G Zheng, Daniel Howsmon, Boliang Zhang, Juergen Hahn, Deborah McGuinness, James Hendler, Heng Ji

**Affiliations:** 1 Department of Computer Science, Rensselaer Polytechnic Institute, 110 8th Street, 12180, Troy, NY, USA; 2 Tetherless World Constellation, Rensselaer Polytechnic Institute, 110 8th Street, 12180, Troy, NY, USA; 3 Department of Chemical & Biological Engineering, Rensselaer Polytechnic Institute, 110 8th Street, 12180, Troy, NY, USA; 4 Department of Biomedical Engineering, Rensselaer Polytechnic Institute, 110 8th Street, 12180, Troy, NY, USA

**Keywords:** semantic web, biological ontologies, text mining, signal transduction, wikification, entity linking, biomedical literature

## Abstract

**Background:**

The Entity Linking (EL) task links entity mentions from an unstructured document to entities in a knowledge base. Although this problem is well-studied in news and social media, this problem has not received much attention in the life science domain. One outcome of tackling the EL problem in the life sciences domain is to enable scientists to build computational models of biological processes with more efficiency. However, simply applying a news-trained entity linker produces inadequate results.

**Methods:**

Since existing supervised approaches require a large amount of manually-labeled training data, which is currently unavailable for the life science domain, we propose a novel unsupervised collective inference approach to link entities from unstructured full texts of biomedical literature to 300 ontologies. The approach leverages the rich semantic information and structures in ontologies for similarity computation and entity ranking.

**Results:**

Without using any manual annotation, our approach significantly outperforms state-of-the-art supervised EL method (9% absolute gain in linking accuracy). Furthermore, the state-of-the-art supervised EL method requires 15,000 manually annotated entity mentions for training. These promising results establish a benchmark for the EL task in the life science domain. We also provide in depth analysis and discussion on both challenges and opportunities on automatic knowledge enrichment for scientific literature.

**Conclusions:**

In this paper, we propose a novel unsupervised collective inference approach to address the EL problem in a new domain. We show that our unsupervised approach is able to outperform a current state-of-the-art supervised approach that has been trained with a large amount of manually labeled data. Life science presents an underrepresented domain for applying EL techniques. By providing a small benchmark data set and identifying opportunities, we hope to stimulate discussions across natural language processing and bioinformatics and motivate others to develop techniques for this largely untapped domain.

## Background

Mining and linking important information from scientific literature can have a tremendous impact on scientific discovery as it is extremely challenging even for domain experts to keep up with the large number of papers published [1]. For example, models of signaling and metabolic pathways are useful tools that aim to concisely represent the known information about a given pathway and accurately predict the effects of different stimuli on cellular processes. Modeling these pathways can aid scientists' understanding of diseases, such as cancer. However, these pathways are very time-intensive to model, usually requiring the human modeler to read numerous papers to obtain the necessary information.

A major bottleneck in understanding scientific literature lies in the enormous amount of unexplained abbreviations and terminologies [2]. For example, the transcription factor "*C/EBP-β*" is also known as "*NF-IL6*"; the protein "*Arnt*" is sometimes referred to as "*HIF1-β*". Being able to identify the key proteins, and their behaviors and interactions, would be extremely helpful for supporting the modeling task. In this paper we focus on the task of Entity Linking (EL) for biomedical literature - automatically identifying prominent entity mentions from unstructured full texts and linking them to (or "grounding them in") terms described in a Knowledge Base (KB) and/or defined in an ontology in order to enrich text documents. These knowledge base or ontology terms are sometimes referred to as reference entities. For example, from the following sentence from Lipniacki et al. [3]:

"In resting cells, **p50-65 heterodimers **(referred herein as **NF-*κ*B**) are sequestered in the cytoplasm by association with members of another family of proteins called **I*κ*B**."

an EL system will identify three prominent mentions "*p50-65 heterodimers*", "*NFκB*" and "*IκB*", and link the first two to "*nuclear factor kappa-light-chain-enhancer of activated B cells*" and the third to "*nuclear factor of kappa light polypeptide gene enhancer in B-cells inhibitor*" in some knowledge base. EL can help human end-users navigate biomedical literature, and improve many other Natural Language Processing (NLP) tasks such as protein-protein interaction event extraction [4,5]. EL is a well-studied problem in news and social media. When we apply state-of-the-art EL techniques to the biomedical domain, we face new challenges. In this paper we will focus on two unique challenges and our solutions to address each of them.

The first challenge lies in the lack of sufficient context for understanding the entity mentions. This requires us to move from non-collective approaches which link each individual mention at a time to **collective inference **by leveraging the global topical coherence and linking a set of relevant mentions simultaneously. The basic idea is that if we know multiple entity mentions are semantically related in the unstructured source texts (i.e., they co-occur in the same sentence, are linked through dependency paths, or play certain semantic roles in the same event, etc.), we can assume they are semantically related and thus their reference entities should also be connected via semantic links in the ontologies. Collective inference is particularly effective to link entities in scientific literature because the authors often assume that the readers are also domain experts with enough background knowledge about these entities.

The second challenge is the lack and the expense of generating labeled EL data for the biomedical domain. Manual EL annotation for a new domain is challenging and time-consuming. Previous EL work mainly exploited Wikipedia as the target knowledge base. Fortunately, there exist many publicly accessible ontologies in this domain such as those in BioPortal [6]. These ontologies contain rich structures with declaratively defined semantic relations, along with comprehensive text descriptions written by domain experts. In this paper, we describe an unsupervised EL algorithm by leveraging well-structured ontologies (e.g., hierarchical and relational structure) and well-defined semantic relations among entities in the ontologies (e.g., subClassOf). Such rich knowledge also enables us to move away from labor-intensive supervised approaches and gear toward a completely unsupervised approach using novel similarity and coherence measures based on graph structures.

There have been extensive studies on extracting entity mentions from biomedical literature (e.g., [2,7-9]). The previous task that is the closest to our study is gene name normalization [10] which focused on linking entity mentions to a list of gene entities [11,12]. Compared to such a list of flat structures, we instead target a broader range of entity types from full texts (rather than only abstracts), and leverage the deeper structures contained in the ontologies.

Although entity mention extraction from biomedical literature has received attention, most of the previous EL work focused on general news and social media domains (e.g., [13,14]). These EL algorithms can be divided into two categories: non-collective and collective inference approaches. Non-collective methods usually rely on prior popularity and context similarity with supervised models [15-17]. Ranking scores for each concept mention are computed individually. Collective approaches further leverage the global coherence between concept mentions normally through supervised or graph-based re-ranking models [18-24]. Collective inference methods address the linking problem through maximizing the agreement between the text of the mention document and the context of the entities of the knowledge base. Graph-base re-ranking models typically collects linking agreement information from training data and propagates the agreement information to other nodes. Both existing non-collective and collective algorithms require large amounts of manually-labeled entity mentions in order to achieve about 85% linking accuracy for the news domain [13,14]. Finally, previous work mainly focused on discovering knowledge from source texts, while limited efforts have been made on exploiting the rich structures of other knowledge bases beyond Wikipedia. DBpedia Spotlight [25] is the only system that leverages Semantic Web data to link entities to DBpedia, a generic dataset derived from Wikipedia.

In this paper, we demonstrate that entropy based collective inference is crucial to acquire and organize deeper knowledge with a higher coverage from the source. Together with our novel utilization of the declaratively defined rich structures in the merged ontologies with comprehensive text descriptions, the whole framework carries rich enough evidence for effective entity linking, without the needs of any labeled data. Specifically, the main contributions of this paper are as follows.

• We propose a new task to link prominent entity mentions in full texts of biomedical literature to rich ontologies.

• We design a novel collective inference approach and build a benchmark for this new task.

• We exploit the rich structures in ontologies to perform EL in a completely unsupervised fashion without any annotation cost, which even significantly outperforms state-of-the-art supervised approaches.

• We provide thorough analysis about the effectiveness of our approach and the remaining challenges, and shed a light on the general research direction of automatic "reading" scientific literature via knowledge enrichment.

## Methods

In this section we will present our EL approach to the biomedical domain. **A more detailed description of this system with applications to other domains is found in** [26].

### Overview

In the discussion that follows, we first define some basic concepts, notations, and preliminary background and then give an overview of the EL system. The entity mentions *m ∈ M *are the prominent phrases in the full text of a scientific paper. We consider all classes, properties, and individuals as described in the ontologies *e ∈ E *to be the reference entities, which are used to ground the entity mentions. Each entity is described by a surface form dictionary that contains all phrases matching its string. For example, the entity "*IKK*" is an entry in *E*, whereas an occurrence of "*IKK*" in a scientific paper is an entity mention. Furthermore, an occurrence of "*IκB kinase*" is one surface form of "*IKK*" because it's a synonym of "*IKK*".

**Figure 1:**
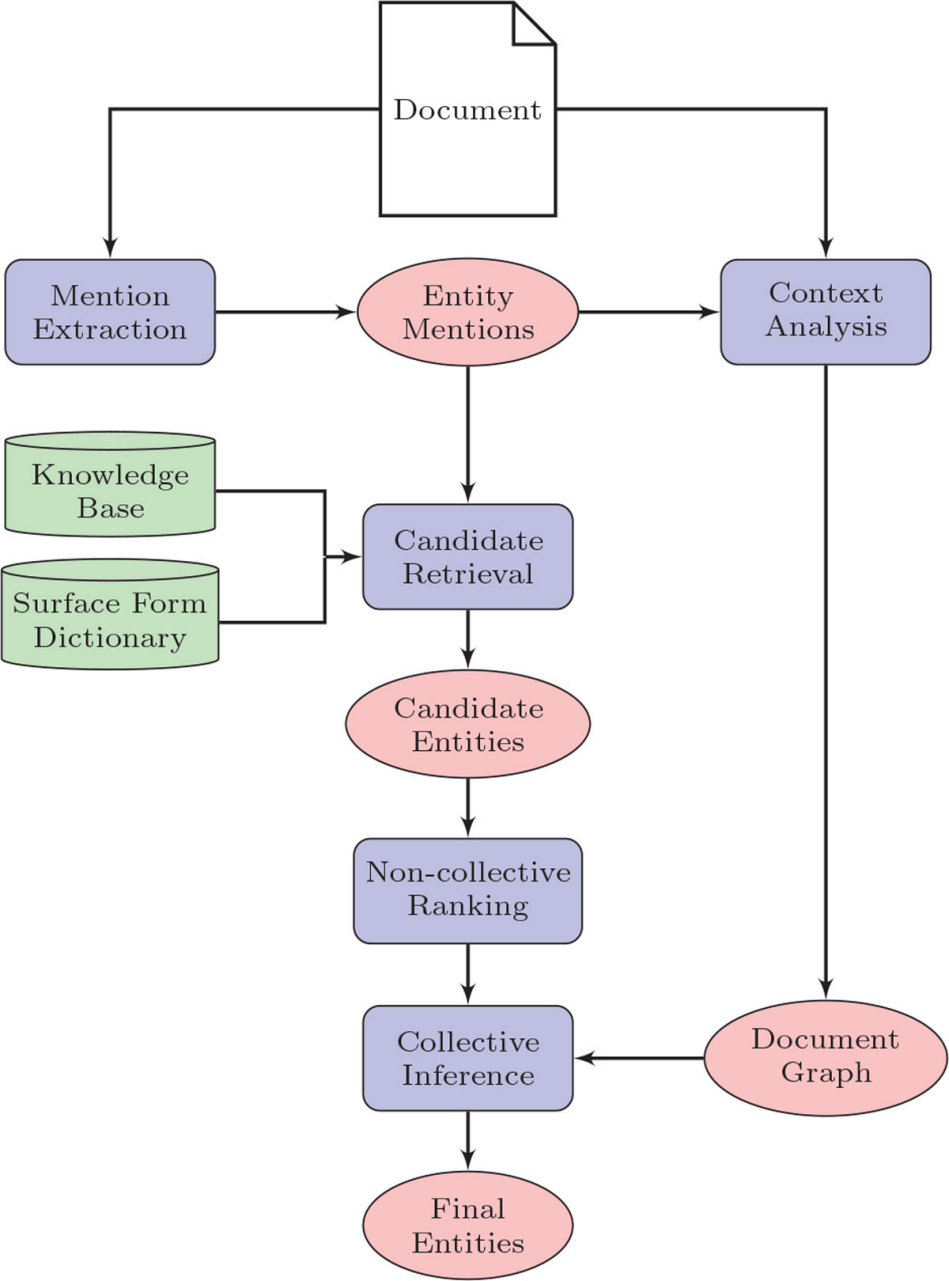
**Approach Overview**. An illustration of the approach described for analysis of a document.

The overall approach is depicted in Figure 1. We first construct a knowledge base (described in the following section ). Next, given a textual document *d*, we extract the entity mentions *M *: {*m*_1_, *m*_2_, ...*m_n_*} as described in section 3.2. We then construct a graph representation *G_d _*= 〈*V, R*〉 for *d*, where *V *= {*v*_1_, *v*_2_, ...*v_n_*} is the set of vertices, each vertex *v *represents an entity mention in *d*, and *R *= {*r*_1_, *r*_2_, ...*r_n_*} is the set of edges. (Note: *G_d _*refers to the graph of document *d *whereas *G_k _*refers to the graph of the knowledge base.) The vertices *v*_1 _and *v*_2 _are connected by an edge denoted as *ε*(*v*_1_, *v*_2_, *r*) if and only if the entity mentions for *v*_1 _and *v*_2 _are related to each other. Here, such a relation is obtained by analyzing the document *d*. For this work, we extract relations based on sentence-level or paragraph-level co-occurrence. Then, for each entity mention *m*, we use the surface form dictionary to locate a list of candidate entities *c ∈ C *for entity mentions in graph *G_d _*and compute an importance score by the non-collective approach detailed in section 3.5. Finally we compute similarity scores for each entity mention/candidate entity pair 〈*m, c*〉 and select the candidate with the highest score as the appropriate entity for linking.

### Knowledge Base graph construction

We utilize a very broad definition of a Knowledge Base (KB). A Knowledge Base is a data set that contains some, potentially limited, structured content along with unstructured content.

Using this broad definition, Wikipedia is a popular knowledge base that is often used for entity linking because it contains structured information such as titles, hyperlinks, infoboxes as well as unstructured texts. However, in order to take advantage of richer structures and domain knowledge which are not offered by Wikipedia, we constructed a knowledge base from 300 biology-related ontologies from BioPortal [6]. Based on the rich structure contained in these ontologies, we created a web of data (WOD). In the WOD, each entity *e *is described as a set of triples *t ∈ T *. For example, a triple (*_:Nucleus, _:PartOf, _:CellComponent *) indicates that the entity "*nucleus*" is "*part of*" the entity "*cell*".

Our expanded knowledge base *E *was constructed using a graph-based approach. *E *consists of classes, individuals, and properties in the aggregated ontologies. Each entity *e *is regarded as a vertex in the knowledge graph *G_k _*. Using our WOD, each entity is connected to other entities via a set of triples *T *. These connections are regarded as the edges of *G_k _*. For example, the entities "*phosphorylating*", "*IKK*", and "*IκB kinase activity*" contained in the GeneOntology [27] are treated as the vertices of our graph. The triples (*_:IκB kinase activity, _:subClassOf, _:phosphorylating *) and (*_:IκB kinase activity, _:relatedTo, _:IKK *) are treated as edges between the vertex "I*κ*B kinase activity" and other vertices in our graph.

### Mention extraction

The focus of the paper is to link identified mentions to the concepts in the knowledge base. Therefore, for identifying prominent mentions from unstructured texts, we apply various publicly available natural language processing tools. First a name tagger [28] is used to extract entity mentions. Regular expressions are used to join named entities that might have been considered separate by looking for intervening prepositions, articles, and punctuation marks. Then, a shallow parser [29] is used to add noun phrase chunks to the list of mentions. A parameter controls the minimum and maximum number of chunks per mention (one and five by default), and whether overlapping mentions are allowed. **Although domain-specific named entity recognition could improve the overall performance of the system, this was not investigated since our focus was on the entity linking problem in this work**.

### Entity candidate retrieval

By analyzing the triples describing the entities, we also construct a surface form dictionary (*f*, {*e*_1 _, *e*_2_...*e_k _*}) where {*e*_1_, *e*_2_...*e_k _*} is the set of entities with surface form *f*. We analyzed the following main properties: labels and names (e.g. rdfs:label), synonyms (e.g. exact synonym from gene ontology), aliases, and symbols (e.g. from Orphanet ontology), providing us with more than 150 properties to construct the surface form dictionary. During the candidate retrieval process, we retrieve all entities with surface forms that are similar to the mentions' surface form, and considered them as candidates for the mentions.

### Non-collective entropy rank

The candidate entities retrieved from the knowledge base are pre-ranked using an entropy-based non-collective approach. The main idea of the algorithm is to assign the entities with higher popularity a higher score. While entities in Wikipedia are universally connected with the same type of link, entities in the ontologies are potentially connected with many kinds of links that may have semantically rich definitions. We can leverage this greater degree of specificity and assign different weights to edges described by different properties. For example, consider the triples (*_: IKK, _:isCapableOf, _:phosphorylation*) and (*_:IKK, _:locatedIn, _:cytoplasm*). Since "*phosphorylation*" and "*cytoplasm*" are connected to "*IKK*" by different relations, we consider their influence on the importance of "*IKK*" to be different.

To capture such differences in influence, we compute the entropy of relations *H*(*p*) [30] as

(1) $$H\left(p\right)=-\underset{o_{p}\in O_{p}}{\sum }\Psi \left(o_{p}\right)\text{log}\left(\Psi \left(o_{p}\right)\right)$$

where *p ∈ P *is a property or relation that has a value *o_p _∈ O_p _*or links to an object *o_p _∈ O_p _*and Ψ(*o_p_*) is the probability of obtaining *o *given the property *p*. The entropy measure has been used in many ranking algorithms to capture the salience of information [31,32], therefore, in our task, we used it to capture the saliency of a property. In the previous example, *p *indicates "*is capable of*" and "*located in*" while *o *indicates "*IKK*" and "*cytoplasm*" respectively. Then *H*("*iscapableof*") and *H*("*locatedin*") are the influence factors between "*IKK*" and "*phosphorylation*", and "*IKK*" and "*cytoplasm*" respectively.

We then compute the salience score of candidate entities using the following non-collective EntropyRank:

(2) $$ER\left(c\right)=\underset{p^{c}\in P^{c}}{\sum }H\left(p^{c}\right)\underset{o_{p}^{c}\in O_{p}^{c}}{\sum }\frac{ER\left(o_{p}^{c}\right)}{L\left(o_{p}^{c}\right)}$$

where *P^c ^*is the set of properties describing a candidate entity *c *and $L\left(o_{p}^{c}\right)$ is number of entities linked to $o_{p}^{c}$. The EntropyRank for each entity starts at 1 and is recursively updated until convergence. This equation is similar to PageRank [33], which gives higher ranks to the popular entities, but we also take the difference of influence of neighbor nodes into consideration.

As described previously, the candidate entities are retrieved from the surface form dictionary based on the above salience measure. Most often, the exact surface form match between an entity mention and a candidate entity cannot be found. However, our rank model allows partial surface form matches with a penalty. Currently we use Jaccard Similarity to compute partial match scores. For example, Jaccard Similarity will be computed for mention "nucleus" and entity "neural nucleus". In the equation below, *JS*(*m, e*) is the Jaccard Similarity score between the surface form of entity mention *m *and the surface form of candidate entity *c*.

(3) $$ER^{*}\left(m,c\right)=JS\left(m,c\right)\cdot ER\left(c\right)$$

### Collective inference

In the non-collective inference approach, each entity mention is analyzed, retrieved, and ranked individually. Although this approach performs well in many cases, sometimes incorrect entity mention/entity links are formed due to the lack of context information. Therefore, we adopt a collective inference approach, which analyzes relations among multiple entity mentions and ranks the candidates simultaneously. For example, given the sentence that contains the entity mentions "*phosphorylating*" and "*IKK*", the collective approach will analyze the two mentions simultaneously to determine the best reference entities.

In Section 3.1, we presented how we construct the document graph *G_d_*. Using the connected *G_d _*and candidate entities retrieved from the non-collective approach, we can compute the similarity between each entity mention *m *from *G_d _*and a candidate entity *c *from *G_k _*. Both *m *and *c *are connected to sets of neighbor nodes, which provide important contextual descriptions for both *m *and candidate entity *c*, respectively. We then use the following equation to compute the similarity score:

(4) $$Sim^{F}\left(m,c\right)=\alpha \cdot ER^{*}\left(m,c\right)+\beta \cdot \underset{p^{c}\in P^{c}}{\sum }H\left(p^{c}\right)\underset{n\in O_{p}^{c}\cap O^{m}}{\sum }ER\left(n\right)$$

Here, $O_{p}^{c}\cap O^{m}$ is the set of neighbors with equivalent surface form between the *G_k _*subgraph for candidate *c *and *G_d _*subgraph for mention *m*. The parameters *α *and *β *are used to adjust the effects of the candidate pre-ranking score and the context information score on the overall similarity score. Based on the optimization results reported by Zheng et al. [26], we empirically set *α *= 15 and *β *= 8 for all experiments. The equation captures two important ranking intuitions: 1. the more popular a *c *is, the higher rank it will be, as captured by *ER*, 2. the more similar between the *G_k _*subgraph for *c *and *G_d _*subgraph for mention *m*, then higher rank will be given to *c*, which is captured by latter part of the equation.

**Figure 2:**
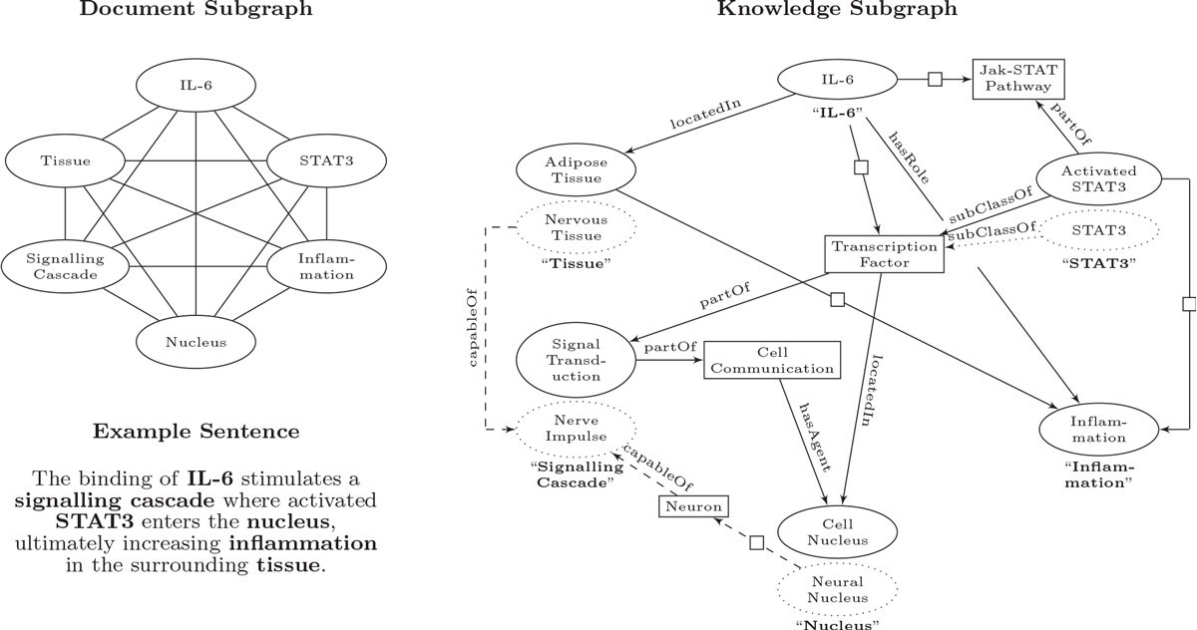
**Illustrative Example**. In the document graph, entity mentions are circled. In the knowledge graph, reference entities are bolded, the candidate entities with the highest ranks are circled with solid lines, and candidates with lower ranks are circled with dashed lines. Boxes indicate intermediates.

To better describe the use of this system for the life science domain, we provide an illustrative example in Figure 2. For the example sentence provided, the document graph *G_d _*has vertices *V *that correspond to entity mentions *M *. For this sentence-level collective inference approach, there exist edges between all vertices since these mentions co-occur in the sentence. We then retrieve our knowledge graph *G_k _*from our knowledge base. Focusing our attention on reference entity "*STAT3*", a term-level search returns candidate "*STAT3*". However, because "*Activated STAT3*" is connected to more vertices of *G_k _*, it is intuitive that this candidate's rank increases with collective inference. Furthermore, although candidate "*Neural Nucleus*" is indirectly linked to "*Nerve Impulse*" which is in turn linked to candidate "*Nervous Tissue*", the isolation of "*Neural Nucleus*" from candidates of other entities enables candidate entity "*Cell Nucleus*" to obtain the highest rank.

## Results

In this section we present the results of our EL method and detailed analysis done by biomedical domain experts.

### Data and scoring metric

To illustrate the use of this approach in the life sciences domain, we analyzed the signal transduction pathway model developed in Lipniack et al. [3]. This paper is extensively cited and backed by a relatively complete set of experimental observations, making it a good candidate for testing our approach. We frequently refer to this reference with the descriptor "Lipniacki" throughout the rest of this paper to avoid ambiguity. **Although this data set is rather limited, there is no known benchmark data for the biomedical domain and one of the advantages of our apporach is that large data sets are not needed for training**. From "Lipniacki", the domain experts in our research team identified 318 mentions of 97 unique prominent entities from 77 sentences and link these mentions to the knowledge base constructed from 300 biology-related ontologies (as described in section ). Among all of the ontologies, there are more than 2 million entities and more than 50 million factual statements. These ontologies were generated and maintained by a combination of domain and knowledge representation experts.

Human annotation focused on nouns and relationships between nouns (e.g. verbs). Nouns were fairly easy to identify for domain-literate persons. Many biological terms have very specific definitions, therefore all entity mentions will have equivalent meanings. For example, "*NF-κB*" is a proper noun referring to a specific protein in a cell. Another example is the term "*transcription*", which refers to the specific process of synthesizing mRNA from a DNA transcript. These situations occur quite often in the nouns annotated. Since the Lipniacki paper is in the primary literature, there were a few terms that were defined explicitly in the paper that are not commonplace in the literature. For example, Lipniacki defines the proper noun IKKa, the activated form of IKK. This author-defined word is easy for a domain-literate person to annotate because the definition is given.

Whereas important nouns were fairly easy to identify, verbs remained a challenge. Some of the verbs have specific definitions. For example, "*phosphorylates*" describes the process of adding a phosphate group to a protein. However, distilling the definition of other verbs was more challenging. For example, the term "*transformed*" as used in the fourth sentence of the Lipniacki abstract refers to a vague process by which IKKn becomes IKKa. This verb is important because it describes a relationship between two terms in the model, but an explicit definition is quite vague due to either incomplete biological knowledge of the process or an attempt by the author to only present the most relevant information for model building.

The mention extraction component associated with both the UIUC Wikifier and our system achieved 63% Precision, 65% Recall and 64% F-Measure. In this paper we focus on developing linking techniques. We use the *linking accuracy *[13,14] to evaluate the linking performance. For each correctly extracted mention, we check whether or not it is linked to the correct entries in the KB.

### Impact of collective inference

To better understand the performance of our wikification system on this new domain, we studied the performance for different inference levels:

1 Mention level: mentions are queried individually and no context information is provided (without collective inference).

2 Sentence level: mentions from the same sentence are analyzed simultaneously (collective inference utilized).

3 Paragraph level: mentions from the same paragraph are analyzed simultaneously (collective inference utilized).

Table 1 presents the results. The improvement from mention level to sentence level illustrates that leveraging the relations among entities presented in the KB via collective inference is beneficial. However, we observe a performance drop from sentence level to paragraph level. By including more mentions, we may potentially introduce unrelated information and noise when compared to the sentence level. For example "*phosphorylating*" was identified correctly at the sentence level, but misidentified at the paragraph level in one example. The broader paragraph level search included terms such as "*NF-κB*, "*signaling pathway*", and "*A20*" which are not connected to "*phosphorylating*" in the aggregated ontologies. There are other examples which were correctly identified at the paragraph level but not at the sentence level, however, these were fewer than those where the sentence level produced an adequate link and the paragraph level did not.

**Table 1:** Result of Collective Inference.

Inference Level	Linking Accuracy
Mention	73.08%
Sentence	83.17%
Paragraph	65.87%

When we are given a single term for disambiguation, we lack context information. The simple popularity-based non collective disambiguation algorithm will always return the most popular referent entity regardless of the context. However, in the biomedical domain, the same mention can refer to different entities in different contexts. On the other hand, collective inference takes advantage of the provided context information during the disambiguation process, which is aligned with the way domain experts disambiguate the terms. For example, the entity "*phosphorylating*" is misidentified at the term level, but is properly identified at the paragraph level. At the mention level, "*phosphorylating*" is identified as "*glyceraldehyde-3-phosphate dehydrogenase (GAPDH)*", a specific protein that carries out a well-studied enzymatic process in cellular metabolism. Furthermore, this protein is responsible for adding a phosphate to a small molecule rather than a protein. However, at the paragraph level, "*phosphorylating*" is correctly assigned to the general process of adding a phosphate group to a protein. In the context of an intracellular signaling cascade, phosphorylating a protein typically alters the protein from an inactive to an active form. Misidentifying "*phosphorylating*" as a specific enzyme (proper noun) rather than a cellular process (verb) may incorrectly state that "*GADPH*" is involved in this signaling cascade and/or miss an important event in the signal cascade, thereby confusing the reader.

At the sentence level, some mentions of "*phosphorylating*" are identified correctly, whereas other mentions are misidentified. For example, in section 2.0 of Lipniacki, "In this form it is capable of **phosphorylating I*κ*B*α***, which in turn leads to its **degradation**."

the system misidentified "*phosphorylating*". In this sentence, since I*κ*B*α *is the object of phosphorylation and GADPH does not perform this phosphorylation, a domain-literate person can readily tell that the definition provided by the algorithm is inaccurate. Furthermore, because 1.) I*κ*B*α *is a protein, 2.) the sentence discusses the actions of phosphorylation or degradation of this protein, and 3.) the queried ontologies do not contain specific entries related to this specific phosphorylation process, it is intuitive to a domain-literate person that the collective inference should help the correct linking of "*phosphorylating*". In the same paragraph of Lipniacki,

"The newly synthesized **I*κ*B*α ***again inhibits **NF-*κ*B**, while **A20 **inhibits **IKK **by catalysing its transformation into another inactive form, in which it is no longer capable of **phosphorylating I*κ*B*α***."

the system correctly identified "*phosphorylating*". In this sentence, since 1.) IKK is a kinase (a protein capable of phosphorylating a specific entity or group of entities), and 2.) I*κ*B*α*, NF-*κ*B, A20, and IKK are all proteins, it is intuitive to a domain-literate person that collective inference would return a correct match.

This relation between "*phosphorylating*" and "*IKK*" is captured and modeled in GeneOntology^[3] ^by biology ontologists. The ontology states that "*phosphorylating*" is related to an activity that involves "*IκB kinase*", a synonym for "*IKK*". Our collective inference algorithm leverages this knowledge during the ranking computation and promotes the initially under-ranked description from GeneOntology to the highest rank when the concept "*IKK*" is presented in the sentence level.

### Comparison with state-of-the-art

To evaluate the performance of our approach, we compare the ontology-based system with [34], one of the current state-of-the-art EL systems trained from news-related data. We compare the linking accuracy scores in Table 2.

**Table 2:** Performance of the Wikifiers

Wikifier	Correct Links	Total Links	Linking Accuracy
(Chan and Roth, 2013)	84	113	74.34%
Our Approach	173	208	83.17%

From the table, we can see that our system significantly outperforms [34] by a wide margin. One way to solve this domain-mismatch problem is to train a Wikifier using a biology-related training dataset. However such a dataset would be expensive and time consuming to generate. For example, the news training dataset used by [34] took a significant amount of time to create and it would be unlikely that this effort would be repeated for a new domain. Furthermore, datasets for a biomedical domain, unlike news-related datasets, require a domain expert with specialized knowledge, which further complicates the task of developing large training sets.

In contrast to this approach, we used biomedical ontologies and a novel unsupervised algorithm for this domain. The advantage of the proposed work is that there are many related ontologies published on the Web by the domain communities such as BioPortal [6]. Since the system relies heavily on the related ontologies, the system performance improves with the quality of the ontologies. Even though generating high quality ontologies is expensive, there are many ongoing efforts to capture and model biology-related knowledge such as the continued work on the Gene Ontology [27]. We can easily leverage these works to improve the system.

### Remaining challenges

Our approach significantly outperforms state-of-the-art without using any labeled data. However, there are several remaining challenges, including:

1 As previously mentioned, our EL system is not able to decide whether or not it returns a link. It is a challenging research question to optimize the threshold to determine whether a mention is linkable or not [35].

2 Failure to detect biomedical concept mentions for linking to the knowledge base constitute about 22% of errors of [34]. Since the biomedical ontologies contain a relatively complete taxonomy dictionary for the domain concepts including synonyms, alias names, and abbreviations, we can leverage this information and apply a dictionary based approach to detect relevant concepts.

3 Although we utilize a large amount of ontologies which capture biomedical knowledge, some facts and relations among concepts are not clearly defined. For example, the fact that "*eukaryotic transcription*" takes place only within the "*cell nucleus*" is not clearly presented. Instead, a vague "*related to*" relation between "*eukaryotic transcription*" and "*cell nucleus*" is presented in the ontologies.

4 Many of the processes taking place in signaling pathways are dependent upon another. For example, it is very common that a protein is phosphorylated which turns it into its active form, which is needed to activate another protein and so forth. As such, there is often a cascade of events that all depend upon each other. While it is known that these types of relationships exist, we currently do not make use of this knowledge.

5 The current disambiguation algorithm assumes that phrases from the same sentence or same paragraph are related to each other, however such assumptions can potentially undermine the EL performance. For example, we observe that including mentions from the same paragraph as context information, our performance drops when compared to only including mentions from the same sentence. Better collaborators for a target entity may be obtained by deep semantic parsing techniques such as Dependency Parsing and Semantic Role Labeling.

## Conclusions

We have developed an effective Entity Linking system to automatically identify and link prominent mentions in unstructured biomedical literature to ontologies. As more and richer ontologies are being constructed and accessible in many scientific domains, we feel the time is now ripe to explore some novel methods to adapt mature text mining techniques to automatically enrich knowledge for scientific papers. By a thorough pilot study, we have demonstrated that it's possible to skip the tedious manual annotation by incorporating rich structures in ontologies in an unsupervised collective inference framework. The proposed approach would save scientists concerned with staying informed about research development an enormous amount of time. In the future, we plan to apply semantic parsing to better select mention collaborators for collective inference, and leverage other existing Semantic Web technologies such as semantic reasoning to improve the linking quality.

## Competing interests

The authors declare that they have no competing interests.

## Authors' contributions

JZ performed experiments related to the presented system and assisted with manuscript preparation. DH evaluated the system performance and assisted with manuscript preparation. BZ compared our approach to a previous supervised approach. JHa supervised the system performance evaluation. DM, JHe, and HJ supervised the experiments.

## Declarations and disclaimer

Publication costs for the article were sourced from the U.S. Army Research Laboratory under Cooperative Agreement No. W911NF-09-2-0053 (NS-CTA), U.S. NSF CAREER Award under Grant IIS-0953149, U.S. DARPA Award No. FA8750-13-2-0041 in the Deep Exploration and Filtering of Text (DEFT) Program, Google Research Award, and RPI faculty start-up grant, DARPA SMISC Program. The views and conclusions contained in this document are those of the authors and should not be interpreted as representing the official policies, either expressed or implied, of the U.S. Government. The U.S. Government is authorized to reproduce and distribute reprints for Government purposes notwithstanding any copyright.

This article has been published as part of *BMC Medical Informatics and Decision Making *Volume 15 Supplement 1, 2015: Proceedings of the ACM Eighth International Workshop on Data and Text Mining in Biomedical Informatics. The full contents of the supplement are available online at http://www.biomedcentral.com/bmcmedinformdecismak/supplements/15/S1.
